# Application of Cavity Enhanced Absorption Spectroscopy to the Detection of Nitric Oxide, Carbonyl Sulphide, and Ethane—Breath Biomarkers of Serious Diseases

**DOI:** 10.3390/s150614356

**Published:** 2015-06-17

**Authors:** Jacek Wojtas

**Affiliations:** Institute of Optoelectronics, Military University of Technology, 2 Kaliskiego Str., Warsaw 00-908, Poland; E-Mail: jwojtas@wat.edu.pl; Tel.: +0048-261-837-943.

**Keywords:** laser absorption spectroscopy, cavity enhanced spectroscopy, CEAS, gas sensors, breath analysis, biomarkers, nitric oxide, carbonyl sulphide, ethane, QCL

## Abstract

The paper presents one of the laser absorption spectroscopy techniques as an effective tool for sensitive analysis of trace gas species in human breath. Characterization of nitric oxide, carbonyl sulphide and ethane, and the selection of their absorption lines are described. Experiments with some biomarkers showed that detection of pathogenic changes at the molecular level is possible using this technique. Thanks to cavity enhanced spectroscopy application, detection limits at the ppb-level and short measurements time (<3 s) were achieved. Absorption lines of reference samples of the selected volatile biomarkers were probed using a distributed feedback quantum cascade laser and a tunable laser system consisting of an optical parametric oscillator and difference frequency generator. Setup using the first source provided a detection limit of 30 ppb for nitric oxide and 250 ppb for carbonyl sulphide. During experiments employing a second laser, detection limits of 0.9 ppb and 0.3 ppb were obtained for carbonyl sulphide and ethane, respectively. The conducted experiments show that this type of diagnosis would significantly increase chances for effective therapy of some diseases. Additionally, it offers non-invasive and real time measurements, high sensitivity and selectivity as well as minimizing discomfort for patients. For that reason, such sensors can be used in screening for early detection of serious diseases.

## 1. Introduction to Laser Absorption Spectroscopy

Optoelectronic methods, which use the phenomenon of optical radiation absorption to detect and measure the concentrations of the molecules, allow achieving low detection limits and high selectivity [1,2,3]. For this purpose, it is necessary to apply radiation, the wavelength of which is matched to the spectral range characterized by strong absorption of the tested molecules, but this task is not so simple, as it is very important to minimize the impact of the absorption spectra of other molecules.

When infrared radiation (IR) of the appropriate wavelength incides on a molecule, a change of the molecule’s vibrational and/or rotational energy occurs (the transitions between electron states correspond to higher energies of photons, the range of the visible and ultraviolet part of the spectrum). The absorption phenomenon is observed at a single narrow spectral line having a typical width (in air) of the order of hundredths of nanometres. Vibrational-rotational states form complex and dense energy level structures, and spectral lines corresponding to transitions between these levels form groups called bands. Generally, the compounds have characteristic absorption bands at certain wavelengths. Based on the knowledge of the shape and distribution of these bands, it is possible to identify molecules. The band corresponding to the smallest change in vibrational energy of the molecule caused by the radiation of the longest wavelength (low energy photons) is called a baseband.

The single absorption line has the shape of a bell curve with a predetermined width. Under the conditions in which the experiments were carried out, *i.e.*, at temperatures close to room temperature, and atmospheric pressure, the most important is the broadening due to the collision of test molecules with air molecules. This can be minimized by reducing the pressure. A relatively small effect is caused by the Doppler effect resulting from the chaotic motion of the molecules. In contrast, of completely negligible importance is the broadening of the spectrum resulting from the widening of energy levels as described by the Heisenberg uncertainty principle (*i.e.*, the product of the energy level broadening and the lifetime of the particles at this level cannot be smaller than *h/4π*, where *h* is the Planck’s constant).

Spectra with clearly separated rovibronic lines can be observed for light particles that are the subject of publications. In the case of complex polyatomic molecules, the oscillation-rotation structure of the spectra is usually very complex, and as a result of broadening the individual lines overlap and a continuous band is observed.

The absorption of optical radiation is usually characterized by the coefficient *α* describing weakening of radiation intensity passing through the medium. This factor can be defined as the inverse of the optical path (expressed in m^−1^), when the radiation intensity decreases *e*-fold. The intensity *I_0_* monochromatic optical radiation of wavelength *λ* after passing through a sample of thickness *l* is reduced to values *I* described by the Lambert-Beer law:

(1) $$I\left(\lambda \right)=I_{0}\left(\lambda \right)e^{-\sigma \left(\lambda \right)Nl}$$

where *N* denotes the sample concentration.

Basing on Equation (1), the detection limit can be expressed in terms of the minimum detectable value of the absorption coefficient (*α_min_*). Then, it is defined by the accuracy of the small changes determination in radiation intensity $\Delta I/I_{0}$, (where $\Delta I=I_{0}-I$). Therefore, fluctuations in the power and in the wavelength of the laser as well as the noises of the photodetector-preamplifier and the measuring unit all have significant influences on the value of *α_min_*. For very low values of *σ(λ)*, the accuracy of this method also decreases. To obtain a low detection limit level, it is necessary to minimize the influence of the noise sources. Despite these treatments, *α_min_* usually reaches a value of about 10^−2^ m^−1^ using the so-called direct detection technique.

A lower detection limit value can be achieved by lengthening the optical path of the radiation , e.g., by the use of multi-pass cells (in a Herriot, White or an astigmatic configuration, *etc.*). Lengthening the optical path is achieved due to multiple beam reflection in multi-pass cells. They provide an optical path 10–100 times longer (or even more). Due to this, it is possible to measure absorption coefficients on the order of 10^−5^ m^−1^ [4]. This detection limit can be decreased to a value of 10^−7^ m^−1^ using the wavelength modulation technique (WM) with phase-sensitive detection procedures.

Another solution is to use optical cavities (resonators), which provide effective optical paths of even up to several kilometres. This enables measuring an absorption coefficient even lower than 10^−9^ m^−1^. For example, this detection limit is achievable in cavity ring-down spectroscopy (CRDS), in cavity enhanced absorption spectroscopy (CEAS) or in integrated cavity output spectroscopy (ICOS) [1]. They use high-finesse optical cavities that consist of two ultra-high-reflectance dielectric mirrors (often *R* > 99.99%). Methods using optical cavities are usually called cavity enhanced spectroscopy (CES) [5].

## 2. Application of CES for Human Breath Analysis

The use of methods providing detection and measuring trace quantities of matter allow early detection of pathological changes. The main advantages of this diagnosis include: non-invasiveness (puncture wounds, placing instruments into the body, use of contrasts, *etc*, are not necessary), ease of use (the storage and transport of samples as well as their preparation for analysis is not needed), reusability, real-time measurement, no additional burden on patients (especially important for children and elderly persons), and easy operation of such devices in comparison to other methods commonly used in medicine.

Currently, there are few techniques applied to exhaled air analyses [6]. In contrast to devices using mass spectrometry (MS), gas chromatography (GC), proton transfer reaction-MS (PTR-MS) or Fourier-transform infrared spectroscopy (FTIR), CES methods have some advantages, e.g., easy operation, high sensitivity without complicated and time-consuming sample preparation procedures, fast and simple diagnostic results, and no need for specialized staff. The CES devices enable continuous measurements that are fully automatic and maintenance-free. They allow the detection and concentration measurement of many type of gases that have absorption spectra in the wavelength range achievable by commercially available lasers.

Due to this, such sensors can be used in medicine for screening patients, controlling the progress of therapy, monitoring exogenous gases and analyzing metabolic gases. In recent years, growing interest in this type of research has been observed. Endogenous and exogenous amino acids occurring in the exhaled air provide information about physiological processes in the human body. The human breath also includes molecules such as H_2_O, CO_2_, O_2_ and N_2_, at a relatively high concentration. In addition, there are over a thousand other components, the concentration of which is in the ppb-ppt (*part per trillion*) range [7]. The presence of specific gases (known as biomarkers) at elevated concentration levels in the exhaled air indicates human diseases. Their concentration depends on the individual characteristics of a person. Examples of selected biomarkers are listed in Table 1.

**Table 1:** Examples of disease biomarkers.

Breath Gas	Formula	Typical Fraction [ppb]	Diseases
Nitric oxide	NO	<35	Asthma, angina, hyperbilirubinemia
Carbon monoxide	CO	1000–10,000	
Pentane	C_5_H_12_	<10	Breast cancer, lung cancer
Aceton	(CH_3_)_2_CO	<1000	Diabetes
Ammonia	NH_3_	<2000	Liver disease, stomach ulcers and duodenal ulcers caused by *Helicobacter pylori*
Carbonyl sulfide	OCS	<10	Liver disease, transplant rejection
Ethane	C_2_H_6_	<10	Alzheimer’s disease, atherosclerosis, diabetes, cancers

Recent progress in optoelectronics provides ways to construct compact and user-friendly sensors designed to detect specific disease biomarkers. The important aspect of CES techniques is to determine the absorption spectra and absorption cross sections of the tested biomarkers. This is closely related to sensor parameters such as the detection limit and selectivity.

## 3. Characterization of Selected Biomarkers

The concentration of specific biomarkers in human breath depends on the individual characteristics of a person or indicate a symptom of a particular ailment. For example, the concentration of nitric oxide is dependent of age, gender, lung capacity and time of day. For women, the concentration is affected by the phase of the menstrual cycle. Moreover, smoking, alcohol consumption and air pollution also have an influence on the breath components [8]. During selection of the absorption spectra of some biomarkers, the main problem was to minimize the influence of other compounds (interferents) especially water and carbon dioxide. Although interferents absorption cross sections at the selected wavelengths are much lower than the biomarkers, their concentration in breath is higher compared to the biomarkers threshold (e.g., concentration of CO_2_ may be five million times higher than the concentration of NO). As a result, the absorption coefficients of water and carbon dioxide are dominant.

### 3.1. Nitric Oxide

Scientists have discovered more and more functions of NO in the body over the past few years, e.g., it is produced in large amounts in inflammatory processes [9]. The nitric oxide level in human breath ranges in volume from several ppb to several hundred ppb. Measurements of fractional exhaled NO (FeNO) provide a new method to monitor the inflammatory status in respiratory disorders, such as asthma and other pulmonary conditions, and in controlling the dose of inhaled corticosteroids, which could provide serious side effects [10].

The American Thoracic Society (ATS) and the European Respiratory Society (ERS) have published recommendations for the online and offline measurement of exhaled lower airway and nasal NO [8]. At present, commercially available FeNO analysers are based on the chemiluminescence technique [1]. Similar sensitivity can be obtained using high-resolution laser absorption spectroscopy. It may provide less frequent calibration, and because of dynamic progress in optoelectronics technology, lower initial and operating costs, and smaller dimensions.

**Figure 1:**
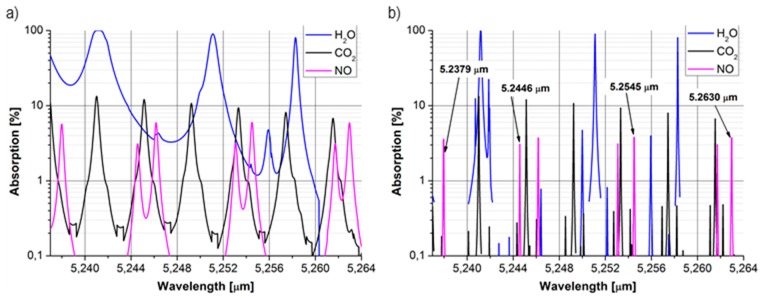
(**a**) The absorption of NO at pressures of 1 atm (**b**) and of 0.1 atm for concentrations occurring in human breath: NO—35 ppb, CO_2_—5%, H_2_O after the drying procedure—279 ppm.

NO has a strong absorption band near a wavelength of 5.26 µm. Figure 1 shows the absorption spectra of compounds which are the most significance interferents of the nitric oxide absorption spectrum.

Reduction of the pressure provides the possibility to select specific absorption lines of NO to avoid interference from CO_2_ and H_2_O. The analysis of the absorption spectra for selected biomarkers was conducted based on the HITRAN database assuming a temperature of 296 K.

### 3.2. Carbonyl Sulphide

Detection of carbonyl sulphide (OCS) is of importance in a number of applications that include not only medical diagnostics, but also atmospheric chemistry. OCS is a potentially toxic agent as a product of industrial procedures. At high concentrations, exogenous OCS can cause central nervous system dysfunctions and even death because of central respiratory depression [11,12]. OCS occurs naturally in exhaled air. Its origin may be related to the oxidative metabolism of carbon disulphide [1]. It has been reported that OCS in healthy human breath ranges from 3 ppt to 30 ppb. Its higher concentration is associated with liver diseases [13].

**Figure 2:**
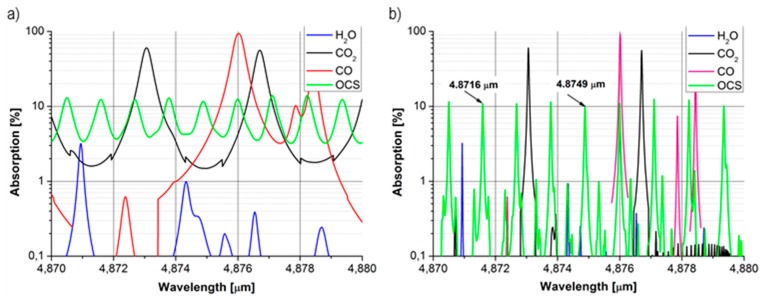
(**a**) The absorption of the OCS at pressures of 1 atm (**b**) and of 0.1 atm for concentrations occurring in human breath: OCS—10 ppb, CO—10 ppm, CO_2_—5%, H_2_O after the drying procedure—279 ppm.

An increased concentration of carbonyl sulphide can be also a signal of lung transplant rejection. Contrary to bronchoscopy, it can be used for non-invasive diagnosis. Such a study was described by Studer *et al.* They observed elevations in exhaled OCS levels in transplant patients with acute rejection compared with stable patients. It is probably caused by tissue necrosis [14]. For people suffering from acute rejection of transplanted lungs, the concentration can even reach 0.5 ppm. The alternative to using laser absorption spectroscopy is either a lung biopsy or gas chromatography and mass spectroscopy analysis lasting up to 4 h [15]. OCS is also known as a biomarker for cystic fibrosis [16]. In Figure 2, some absorption peaks of OCS in the mid-infrared spectrum and its most significant interferents are presented.

### 3.3. Ethane

It has been observed that exhaled ethane concentration is elevated in lung diseases such as chronic obstructive pulmonary disease (COPD), asthma, cystic fibrosis (CF), and acute respiratory distress syndrome (ARDS) [17,18,19,20]. Increased levels of ethane are also related to oxidative stress (OS) in some forms of cancer [21,22], heart transplant rejection [23], and bronchial asthma [22]. Generally, OS can be associated with critical disease or contributing to organ failure [19].

Although there are other potential sources of hydrocarbons in the body, such as protein oxidation and colonic bacterial metabolism, in practice they do not interfere with the interpretation of the hydrocarbon breath test for ethane [24]. Hydrocarbons as stable final products of lipid peroxidation show only low solubility in blood, but they are exhaled within a few lung passages. Hence, these compounds can be used as fast markers of oxidative damage in the body. In addition, the severity of injury may be estimated from the relative increase of exhaled ethane or pentane concentrations. For example, treatment with vitamins *E* and *C* as well as a low-fat, high-vegetable diet significantly decrease exhaled ethane and pentane. Smoking, on the other hand, is associated with the opposite effect [25].

Pentane can be accumulated in human fat deposits, from which it is then released slowly over a period of several days [26], which is why its levels may not be directly related to events just prior to breath collection. By contrast, ethane is highly volatile, and measurements of breath ethane may provide an indicator of oxidative stress that directly correlates to the current physiological state of the patient [27].

**Figure 3:**
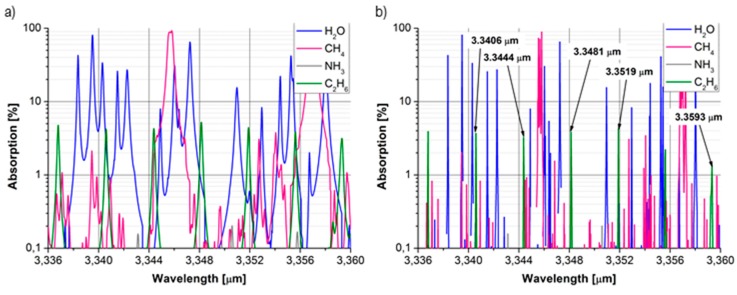
(**a**) The absorption of C_2_H_6_ at pressures of 1 atm (**b**) and of 0.1 atm for concentrations occurring in human breath: C_2_H_6_—10 ppb, NH_3_—2 ppm, CH_4_—1.7 ppm, H_2_O after the drying procedure—279 ppm.

The study of volatile organic compounds (VOCs) in the exhaled air of lung cancer patients usually includes C_4_–C_20_ hydrocarbons [28,29]. Hydrocarbons at the concentration range of ppb‒ppt are often determined using gas chromatography coupled to flame ionization or mass selective detection [30]. To detect ethane (C_2_) at ppb levels, GC-based systems require pre-concentration of the tested air. The time necessary to measure a single sample is typically more than half-an-hour [19]. Similar results can be obtained much faster using cavity enhanced spectroscopy. A low detection limit can be obtained by the appropriate selection of the ethane absorption peak (Figure 3).

## 4. Experimental Results

The CES applicability to detect biomarkers in human breath was studied using a PG711-DFG-SH tunable laser system from the Ekspla Company (Vilnius, Lithuania) and a sbcw1517 quantum cascade laser from Alpes Lasers SA (Neuchâtel, Switzerland). The laser system allows laser pulses with a time duration of 70 ps and energy of 37 µJ (at 5.2 µm). It allows one to tune the wavelength in the 1.55–2.02 µm and 2.25–16.0 µm ranges. Because of its spectral parameters (limited tuning resolution of 1 nm) and a broad laser linewidth at the wavelengths of interest, the efficient matching of this laser system spectrum to the absorption spectra of the tested gases was not possible. In addition, the application of this system (or a similar one) is not practical because of its complicated construction and high operating condition demands.

**Figure 4:**
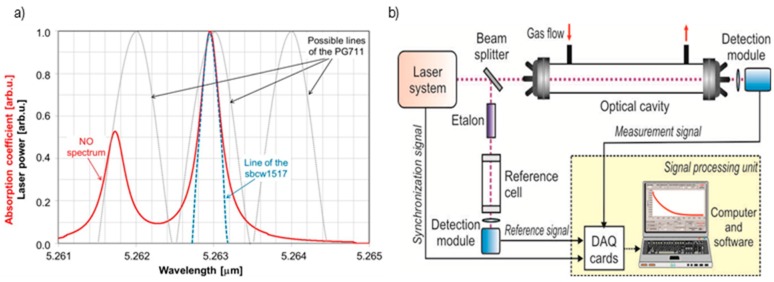
(**a**) Comparison of lasers linewidths and the nitric oxide absorption spectrum; (**b**) and block diagram of the experimental setup.

These restrictions do not exist in the case of single-mode quantum cascade lasers. The QCL linewidth is much narrower. It is mainly limited by the noise of the power supply and driving systems [31]. As shown in Figure 4a, the emission lines of the QCL can be very well matched to the width of a typical molecular absorption spectrum (calculation of the nitric oxide absorption coefficient was performed for the temperature of 296 K and a pressure of 1 atm; laser parameters were adopted on the basis of the manufacturer’s data). Due to this, efficient absorption measurements are possible. The applied laser allows wavelength tuning with high precision in the 5.248 µm (at a temperature of −20 °C) to 5.274 µm range (at a temperature of 30 °C). It offers 13 mW average power under the conditions presented in Table 2.

**Table 2:** Example of the QCL operating parameters.

Lasing Wavelength	5.2627 µm
Voltage supply	10.452 V
Average current	0.095 A
Laser temperature	2.8 °C
Duty cycle	40% (@1 kHz)
Beam diameter	3 mm
Beam divergence	<3 mrad

In the detection system the CEAS configuration was applied (Figure 4b). Thanks to this, extremely high sensitivity (ppb level), selectivity and real time measurements (<3 s) were obtained. The system consists of a laser system, optical cavity (60 cm length and 0.6 litre volume), detection module, and a signal processing unit including data acquisition cards and a computer. A similar approach has been already described in our previous publications [32].

In order to conduct QC laser wavelength monitoring, a reference cell (3 cm length and 0.85 cm diameter), a germanium etalon and a MCT detection module were used. Because the QCL is very sensitive to electrical surges and instabilities, a high quality power supply was used in the laser system (E3634A, Agilent, Santa Clara, CA, USA). For pulse mode operations, the pulse generator (DG645 type, Stanford Research Systems, Inc., Sunnyvale, CA, USA) connected to the laser driver (LDD400, Alpes Lasers) was applied. Due to careful control of laser temperature (using temperature controller TCU 200, Alpes Lasers) and current, it was possible to precisely tune the lasing wavelength to the selected absorption lines of the investigated gas.

During the experiments with QCL, an optical cavity built of two mirrors (made by VIGO SL, Ożarów Mazowiecki, Poland) with a reflectivity of 99.5% was used. In the case of experiments with the tunable laser system, the optical cavity was equipped with mirrors (CRD Optics Inc., Mountain View, CA, USA), the reflectivity of which was much higher—99.98%. This was possible due to the much higher power of the applied laser and the higher value of SNR on the output of the detection module.

Optical radiation at the output of the optical cavity and at the reference cell output were registered using optimized detection modules of VIGO System S.A. The main elements of the modules were MCT photodiodes, transimpedance preamplifiers and TEC units [33]. Next, voltage signals from the modules were digitized and transmitted to the computer using two data acquisition units (CS328, Cleverscope Ltd., Epsom, Auckland, New Zealand). Analysis of the signals, averaging procedure and determination of gas concentration were performed with special software.

Determination of the wavelength values during laser retuning was performed with the reference cell and etalon. The cell was filled with a test gas of high concentration. Due to the high precision and good stability of the QCL driving system, high resolution of wavelength retuning was achieved. It amounted to 0.0034 nm in the case of current tuning and 0.0462 nm for temperature tuning.

The determinations of tested gas concentrations were based on a two-step process. First, the decay time *τ_0_* of lasers radiation in the optical cavity without an absorber was determined. Then, the cavity was filled with the absorbing mixture and the respective decay time values *τ_1_* were measured. Knowing the absorption cross section σ of the compound, its concentration was calculated from the formula:

(2) $$N_{x}=\frac{1}{N_{o}c\sigma }\left(\frac{1}{\tau_{1}}-\frac{1}{\tau_{0}}\right)$$

where *N_o_* denotes the Loschmidt’s number, while *c* is the light speed. Detailed explanation of the formula can be found in [34].

Detection limit can be determined from the following equation:

(3) $$DL=\frac{\delta_{\tau }}{c\sigma \tau_{0}}$$

where $\delta_{\tau }$ denotes decay time measurement uncertainty.

**Figure 5:**
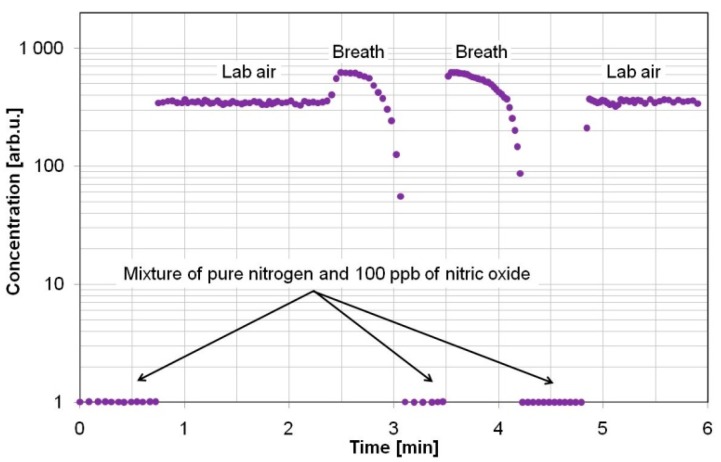
Example of the NO concentration measurements in human breath at atmospheric pressure.

The results of the preliminary experiments are presented in Figure 5. They were normalized to the concentration measurements of a pure nitrogen mixture containing 100 ppb of nitric oxide to show the errors caused by interfering molecules. During nitric oxide concentration measurements, a sensor with QCL was used. The samples were delivered to the sensor at atmospheric pressure and at a temperature of 296 K, and without any drying procedure. First, concentration measurements of 100 ppb NO mixture were carried out under a gas flow of 0.5 L/min. Measurements with lab air samples delivered to the cavity with a flow of 5 L/min show the nitric oxide concentration was much higher than the normal level (in the lab air it should be below 1 ppb). It was caused by absorption of water molecules (Figure 1a). The water influence on measurements was more than 99.9%. The last observation consisted in NO concentration measurements in breath samples (the flow was not continuous and was not controlled). In this case, the results were also much higher than the expected value (the concentration should be below 35 ppb). For breath analyses, a strong absorption of the laser beam by the water and carbon dioxide molecules was observed. The estimated impact of these molecules was 99.86% and 0.13% respectively. Different rise and fall times of the results after changing of the gas source were caused by different values of gas flow.

The observed influence of interferents justified the precise selection of the biomarker absorption lines. To avoid high uncertainty of the measurements and to ensure an appropriate selectivity level, in the subsequent experiments the pressure was reduced and the drying procedure was applied.

During the investigations, concentration measurements of reference gas mixtures were also carried out. The mixtures were prepared as biomarker signatures using the 491 M type gas standards generator from KIN-TEK Laboratories Inc. (La Marque, TX, USA). The high precision of the generator from a level of part per trillion to the initial concentration of 1:1 is guaranteed. The instrument also produces both dry and moistened standard gases, which can be supplied to the sensor at an adjustable pressure. During experiments the KIN-TEK trace source permeation tubes presented in Table 3 were used.

**Table 3:** Type of applied permeation tubes and achieved gas concentrations.

Gas	Type of Permutation Tube	Achieved Concentrations	Comments
NO	57SA 4316	1 ppb *–2 ppm **	Refillable tube
OCS	ELSRT2W 45184	1 ppb *–270 ppb **	Disposable tube
C_2_H_6_	57HA 4573	5 ppb *–6.9 ppm **	Refillable tube

* Using the following KIN-TEK modules: 491MB, 491M-GF and 491M-SD; ** Using the following KIN-TEK modules: 491MB, 491M-GF.

**Figure 6:**
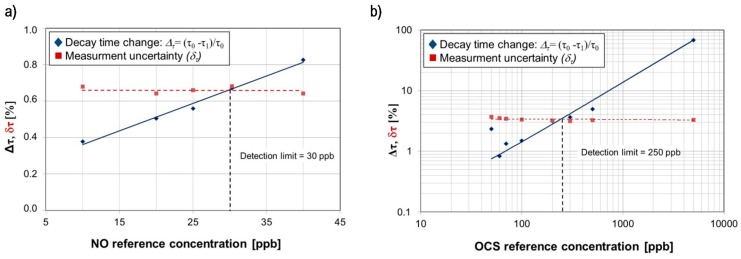
(**a**) The decay time changes of radiation in the optical cavity filled with reference concentration of NO (**b**) and OCS registered in the CEAS setup equipped with the tested QCL.

Experimental setup employing QCL provides detection limits of 30 ppb for NO and 250 ppb for OCS (Figure 6). During experiments QCL operation conditions were following: pulse duration 13 ns, pulse repetition frequency 1 kHz, laser driver voltage U = 10.454 V (average current < 1 mA), laser temperature 1.7 °C and 10.4 °C in case of experiments with OCS and NO respectively. Measurements uncertainty was determined as a standard deviation of the mean value of the decay time. According to the ATS recommendation, high NO concentration levels, *i.e.*, >50 ppb for adult patients and >35 ppb for children, can be caused by atopic asthma, eosinophilic bronchitis or COPD with mixed inflammatory phenotype.

The OCS measurement results show that the detection limit was determined by both the tested QCL parameters and a low absorption cross-section at the applied lasing wavelength. Theoretical analysis defined the possibility to reach a detection limit of 0.9 ppb. It can be done by application of a 10-fold powered QCL with the emission wavelength adjusted to the OCS absorption line characterized by a high value of the absorption cross section (e.g., 4.875 microns) and an optical cavity with a higher quality factor.

The task of the next experiments was to determine the detection limits for ethane and carbonyl sulphide using the tunable laser system. In the case of radiation with a wavelength of 3.3481 µm, the system provides a decay time *τ_0_* of 2.6 µs and a 0.15% time measurement uncertainty. Assuming an ethane absorption cross-section of approx. 2.3 × 10^−18^ cm^2^, a detection limit of 0.3 ppb was obtained (Equation (3)). When the PG711 system was tuned at a wavelength of 4.8716 µm (OCS absorption line), close to the maximum of the mirrors reflection coefficient, a decay time *τ_0_* of 6.1 µs and a time measurement uncertainty of 2.6% were obtained. According the HITRAN database, the OCS absorption cross-section at this wavelength can be ~6.3 × 10^−18^ cm^2^. Based on the Equation (3), the detection limit of 0.86 ppb was achieved.

**Figure 7:**
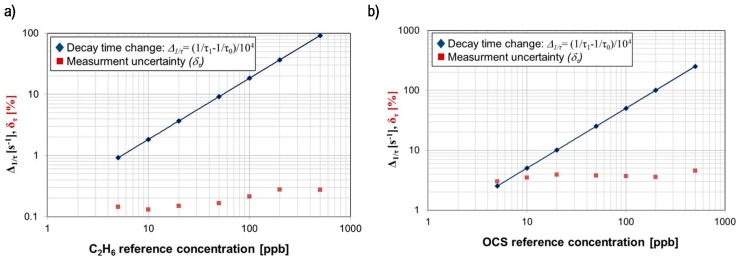
(**a**) Example of measurements results for ethane reference samples (**b**) and for OCS reference samples registered in the CEAS setup equipped with the PG711-DFG-SH system.

Examples of concentration measurements performed for ethane and carbonyl sulphide reference gas samples are presented in Figure 7. They show good linearity and low uncertainty. Due to the limitations of the delivery gas system, it was impossible to carry out the investigation with a lower concentration. Comparing the detection limit of carbonyl sulphide, a considerably better result was obtained in that setup. This was due to the broader range of spectral tuning, the higher power of the laser and the longer optical path obtained by application mirrors with higher reflectance.

The obtained detection limits of gases related to selected diseases biomarkers with the use of designed CEAS sensors are summarized in Table 4.

**Table 4:** Summary of achieved detection limits for selected biomarkers.

Wavelength [µm]	Biomarker	Detection Limit [ppb]	Uncertainty [%]	Type of Applied Laser
5.2630	NO	30	0.7	QCL
5.2624	OCS	250	3.4	QCL
4.8716	OCS	0.9	2.6	Tunable laser system
3.3481	C_2_H_6_	0.3	0.2	Tunable laser system

## 5. Conclusions

Disease diagnosis based on breath analyses still requires sophisticated equipment and excellent skills. Recent developments and technological progress in optoelectronics provide the possibility to improve and simplify such analytical techniques for routine use in the near future. Real-time investigation of volatile substances at the ppb range is possible using laser absorption spectroscopy. Experiments performed demonstrated that CEAS sensors can be effectively used for ethane detection. In the future, such devices will find application in, e.g., screening for early cancer detection. Data of the National Cancer Registry in Poland shows that this kind of study may affect more than 6.4 million men (lung cancer) and approximately 4.8 million women (breast cancer). Such a large demand for screening significantly exceeds the current capabilities of the national healthcare system. It should also take into account the fact that this problem is growing every year. Therefore, it is extremely important to develop sensitive and relatively cheap sensors that could be easy used in health clinics or doctors’ offices.
